# EPR Imaging of Metallic Lithium and its Application to Dendrite Localisation in Battery Separators

**DOI:** 10.1038/s41598-018-32112-y

**Published:** 2018-09-25

**Authors:** Arvid Niemöller, Peter Jakes, Rüdiger-A. Eichel, Josef Granwehr

**Affiliations:** 1 Forschungszentrum Jülich GmbH, Institut für Energie und Klimaforschung (IEK-9), 52425 Jülich, Germany; 2 Institut für Physikalische Chemie, RWTH Aachen University, 52056 Aachen, Germany; 3 Institut für Technische und Makromolekulare Chemie (ITMC), RWTH Aachen University, 52056 Aachen, Germany

## Abstract

Conduction Electron Paramagnetic Resonance Imaging (CEPRI) is presented as a sensitive technique for mapping metallic lithium species. The method is demonstrated using different samples that are either thick or thin compared to the microwave skin depth. As a thin sample, microstructured metallic lithium deposits in a lithium-ion battery (LIB) separator were analysed, illustrating the capabilities of CEPRI by obtaining a high-resolution image with an image resolution in the micrometre range. Limitations and intricacies of the method due to non-linear effects caused by the skin effect are discussed based on images of surface patterns on thick metallic lithium samples. The lineshape of the EPR spectrum is introduced as a proxy to determine the suitability of CEPRI for the quantitative visualisation of metallic lithium deposits. The results suggest that CEPRI is particularly suited to analyse the spatial distribution of microstructured Li that forms during charging and discharging of LIB cells, including the localization of the point of failure in the case of an internal cell short circuit caused by dendrites.

## Introduction

With its high theoretical specific capacity of 3860 mA h g^−1^ and the low negative electrochemical potential of −3.04 V *vs*. the standard hydrogen electrode, metallic lithium is considered an attractive anode material option for rechargeable lithium-ion batteries (LIBs)^1^. Despite these desirable properties, a practical application in lithium-ion cells is hampered by a hitherto non-controllable formation of lithium dendrites^2^ and a limited Coulombic efficiency during Li deposition/stripping^1^. To enable the safe and efficient operation of lithium metal anodes, a fundamental knowledge of the underlying mechanisms that promote dendritic lithium growth and change the metallic lithium anode surface when depositing lithium is indispensable. Imaging methods are desirable that are non-destructively and non-invasively monitoring lithium anode surfaces and dendrite growth.

Comparing imaging techniques capable of visualizing metallic lithium, each of them manifests different advantages and disadvantages. Electron microscopy is a high-energy surface analysis method providing high resolution images, but potentially leading to radiation damage^3,4^. Optical methods such as light microscopy are widespread for image recording with relatively high resolution^5^, but are generally limited to display the surface only. Surface imaging modalities are difficult to interpret quantitatively and, furthermore, only provide a limited insight into, *e.g*., dendrite growth and distribution in separators and solid electrolytes. Computer tomography (CT) measurements allow high resolution imaging and are not surface limited, but need to be performed with an optimal radiation power to avoid radiation damage^6,7^. Consequently, highly optimized synchrotron measurements are necessary for two- and three-dimensional imaging of delicately structured metallic lithium.

To avoid sample radiation damage, low energy imaging methods are preferable. Nuclear magnetic resonance imaging (MRI) has proven to be a suitable non-invasive complement to traditional imaging techniques, with applications as diverse as medical diagnostics, materials sciences, biology or earth sciences^8^. It was also demonstrated to be capable of lithium microstructure imaging, either directly^9,10^ or indirectly^11,12^. While its application is not limited to surfaces, resolution is lower than with optical or electron microscopy techniques. In fact, resolutions below 100 µm are hard to obtain for Li^13^.

Electron paramagnetic resonance (EPR) spectroscopy is more sensitive than nuclear magnetic resonance, while still employing low energy radiation that does not affect the chemical properties or the morphology of the investigated samples. Conduction EPR (CEPR) is well established for the detection of conduction electrons in metallic lithium^14,15^. Some recent lithium CEPR work focused on the state-of-charge dependent, *in operando* identification of mossy lithium in a LIB^2^ and the quantitative analysis of lithium plating on graphite^16^. Both applications are very challenging with alternative techniques and demonstrated the complementarity of CERP compared to more widespread analysis techniques for electrochemical systems. It was shown that the Li morphology could be distinguished in a straightforward manner by the CEPR linewidth of the metallic lithium signal, with porous lithium showing a much narrower EPR line than bulk lithium. Therefore, CEPR is a sensitive indicator for the dimensions of the lithium structures.

Spin density mapping with electron paramagnetic imaging (EPRI) is mostly used for non-conductive, paramagnetic samples, often in combination with spin probes^17,18^. As solely the electron spin is detected, paramagnetic components can be selectively visualized in sample composites, which may considerably simplify the image and its interpretation. However, EPRI is not as widespread as nuclear MRI. Due to the very short relaxation times of electron spins compared to nuclear spins, Fourier EPRI using pulsed field gradients is rarely employed^19^. Instead, projection reconstruction with static magnetic field gradients along different directions in combination with continuous wave (CW) excitation is used.

Sathiya *et al*. qualitatively demonstrated the feasibility of lithium EPRI in a LIB, yet the data processing procedure and filters they used disrupted the quantitativity of the result^20^. Their system was particularly challenging due to the simultaneous presence of multiple EPR-active species – one of which was metallic Li – showing different signal phases. Such differential phase shifts, which are inherent to CEPR experiments, lead to artifacts in the image signal intensity and cause spurious features such as apparent holes. For quantitative imaging, data processing would need to be adapted to the particular characteristics of the sample. As a solution, they suggested to record a full spectrum for each pixel in the image, yet a single experiment would take five days to complete.

In general, EPR investigations of conductive samples are influenced by the skin effect, which leads to a depth dependent attenuation as well as a sample thickness dependent phase shift of the EPR signal^21^. If the sample thickness *d* is larger than the skin depth $\delta$ at the frequency used for spin excitation, which is about $\delta$ ≈ 1.1 µm for metallic lithium at X-band microwave frequencies, the experimentally acquired first derivative EPR spectrum shows a Dysonian lineshape that can be approximated by a phase-shifted Lorentzian^14,21^. For CEPRI, it has been demonstrated that this effect can influence results and additional measurements with positive and negative gradients are necessary for complete data sets^22,23^. In the case of very thin conductive samples, *i.e*. $d\ll \delta$, a Lorentzian lineshape with, at most, a very small phase shift is obtained. Thereby, quantitative imaging is possible since all electrons spins are contributing to the EPR signal^16^. If *d* is increased and exceeds $\delta$, the microwave phase shifts with growing thickness until the case of a thick sample is reached.

The resolution achievable in an EPRI experiment can be discussed using the same concepts as for MRI^24,25^. Formally, a common definition consists of the full width at half maximum of the point spread function^26^. In EPRI without further image processing, the EPR linewidth in the absence of a magnetic field gradient and the applied imaging gradient determine the image resolution. Then, the pixel or voxel length ${\rm{\Delta }}z\,$of the EPR image is proportional to the EPR peak-to-peak linewidth ${\rm{\Delta }}{H}_{{\rm{pp}}}$ and to the inverse of the gradient *G*^27^,

1 $${\rm{\Delta }}z\propto \frac{{\rm{\Delta }}{H}_{{\rm{pp}}}}{G},$$

*i.e*. narrower lines and stronger gradients allow for higher resolution images. However, the magnetic field produced by the gradients cannot be increased arbitrarily. First of all, once the number of spins within a pixel or voxel decreases below the detection limit, no image can be acquired any more. In addition, to prevent disturbing the imaging result, the field produced by the gradients at any point in the sample needs to be much weaker than the static magnetic field *B*_0_ to fulfil the linear gradient approximation. Otherwise concomitant field effects have to be taken into account, which considerably complicates image processing^28^.

Another resolution limiting factor, which is inherent to conduction EPRI yet analogous to imaging of liquids and gases, is the mobility of the spin carriers^29^. Since the spin-carrying electrons are mobile, their position can only be located with a resolution corresponding to the lengthscale sampled by the spins within a representative time. With CW detection, the spin–spin relaxation time constant ${T}_{2}$ of the electrons represents this time, hence the resolution along a particular dimension $z$ is limited to the spin depth^15^,

2 $${\delta }_{e}=\sqrt{2{D}_{z}{T}_{2}}$$

where ${D}_{z}$ is the self-diffusion coefficient of the conduction electrons. ${\delta }_{{\rm{e}}}$ represents the distance a spin-carrying electron covers on average during ${T}_{2}$. For bulk metallic Li, this is on the order of ${\delta }_{{\rm{e}}}\approx 60\,\mu {\rm{m}}$^30^. However, if electron motion is hindered, *e.g*. caused by the morphology of the conductor or between electronically disconnected parts of the sample, or if ${T}_{2}$ is reduced due to impurities in the metal^31^, this resolution limit improves^32^. As a special case potentially relevant for dendritic Li, if different metallic Li pieces are electronically disconnected, then each fragment can be resolved with a resolution corresponding to its size, yet features within a fragment are resolved only down to a lengthscale limited by ${\delta }_{{\rm{e}}}$.

If the sensitivity of the data is high enough, reference deconvolution can be employed for resolution enhancement by reducing the EPR linewidth during post-processing^33,34^. In this case, the intrinsic image resolution ${\rm{\Delta }}z\,$can be estimated by replacing ${\rm{\Delta }}{H}_{{\rm{pp}}}$ in eq. (1) by the filter bandwidth of the window function used for the deconvolution^34^. Eventually, the achievable resolution depends on the sensitivity of the detector^35^.

Moreover, CW EPRI by default records the first derivative spectrum, yet to obtain an image the absorption data is needed. As long as the EPR spectrum is spatially homogeneous, reference deconvolution can also be used to obtain individual sample projections from the first derivative data^36^. In detail, let *S* be the absorption EPR spectrum and *p* the projection of the signal onto the direction of the applied magnetic field gradient. Then the experimentally recorded spectrum is $(S\ast p)\text{'}$, where $(\ast )$ represents the convolution and the apostrophe the first derivative with respect to *B*_0_. Since

3 $$(S\ast p)\text{'}=S\text{'}\ast p$$

applies for the first derivative of a convolution, *p* can be recovered by deconvolution with *S*′. However, *S*′ is not a priori known. A practical solution is to use the EPR spectrum recorded without external gradients as an estimate for *S*′. Therefore, the EPR spectrum must be the same everywhere in the sample. For CEPRI of lithium metal, reference spectra should only contain one component. If this condition is met, then reference deconvolution also corrects a potential effect on image reconstruction caused by a phase shift in the EPR spectrum.

The ability to fit the EPR signal with a single, possibly phase-shifted Lorentzian can be taken as an indication that only a single Li species is detected by CEPR, implying that CEPRI is a suitable imaging modality for a particular sample. The presence of multiple Li species, *e.g*. with different purity, morphology or thickness, substantially complicates a quantitative or semi-quantitative analysis of the CEPR spectrum^2^ and the signal may become non-linear altogether^37^. In this case, CEPRI as demonstrated here is a qualitative technique at best.

In this contribution, conduction EPR imaging is demonstrated as a non-invasive analysis technique for single component systems with different lithium morphologies and thickness. Thick samples were investigated in terms of surface patterns, and the origin of the image contrast in this case is discussed. For a battery with a Li metal anode, fine metallic lithium structures found post-test in the separator are imaged. Thereby quantitative Li metal densities were obtained, irrespective of visibility on the surface.

## Results and Discussion

### CEPR Spectra

**Figure 1:**
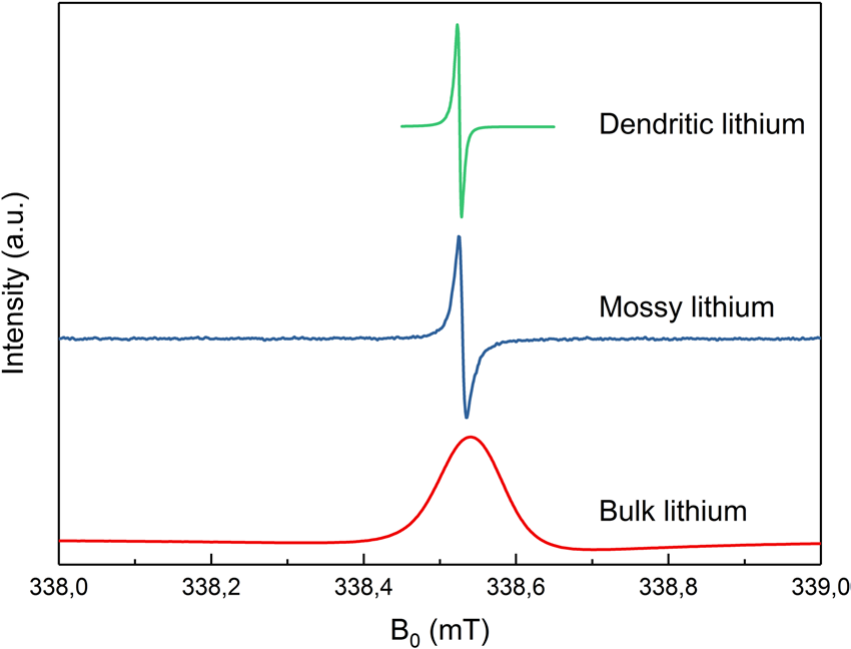
First derivative CEPR signal, as measured in a field swept EPR experiment, for metallic lithium with different morphologies. The peak-to-peak linewidth is minimal for dendritic lithium with ca. 0.005 mT (green), showing a Lorentzian lineshape. For mossy lithium it increases to 0.03 mT (blue). It reaches a maximum for bulk lithium with ca. 0.15 mT (red), showing a Dysonian lineshape.

In Fig. 1, the CEPR spectra of metallic Li with different morphologies is shown. Thick lithium, with a skin depth $\delta \ll d$, shows a typical Dysonian lineshape with a peak-to-peak linewidth of ca. 0.15 mT. Mossy lithium that is formed during electrochemical cycling of a battery with metallic Li anode has a linewidth of ca. 0.03 mT. Finally, dendritic lithium in a battery separator shows a linewidth of ca. 0.005 mT.

Not only the linewidth but also the lineshape depends on the lithium morphology. For dendritic lithium, a Lorentzian lineshape with a ratio of maximum to minimum peak intensity of nearly –1 is obtained, implying that the dimensions of the metal are considerably below the microwave skin depth and that shielding effects are not dominant, hence image intensities can be interpreted quantitatively in terms of local spin density (or concentration). In combination with the narrow lineshape this also indicates that only a single metallic Li species is contributing to the signal and that all lithium structures are dendritic, *i.e*. no bulk lithium was formed or sticking to the separator. Therefore, CEPRI is ideally suited to study dendritic lithium as it combines a narrow linewidth, facilitating a high resolution, and a Lorentzian lineshape that simplifies data recording and analysis, even in a quantitative manner.

The CEPR lineshape of mossy lithium is also Lorentzian, yet it already shows a slight phase shift. The microwave field sees the effective thickness of the mossy lithium, which may be approximated as the sum of the metal thickness through the porous sample^38^. Even if the sample size is larger than the microwave skin depth, the effective thickness can be only slightly higher than the skin depth, resulting in a small phase shift^14^.

The thick sample resulted in a Dysonian lineshape. As only one signal component was found, no significant surface changes, for example caused by surface oxidation, are present. In case of significant chemical surface modifications, two EPR lines would be expected^2^.

Qualitatively, this linewidh change can be conceived as a consequence of self-diffusion of spin-carrying conduction electrons. Since the spin depth is much bigger than the skin depth, ${\delta }_{{\rm{e}}}\gg \delta$, electrons diffuse from the surface into the bulk of the material with a time constant that is considerably shorter than ${T}_{2}$. Thereby excited electron spins are withdrawn from a region excited by the external microwave field and replaced by electrons with an equilibrium spin state^15^. From the point of view of the exciting microwave, this represents an effective acceleration of relaxation, leading to a line broadening. Therefore, as long as only a narrow dendritic line is observed, all the spins are contained in structures that are fully penetrated by the microwave field, hence quantitative imaging of the spin density is possible.

### CEPRI of thick sample

**Figure 2:**
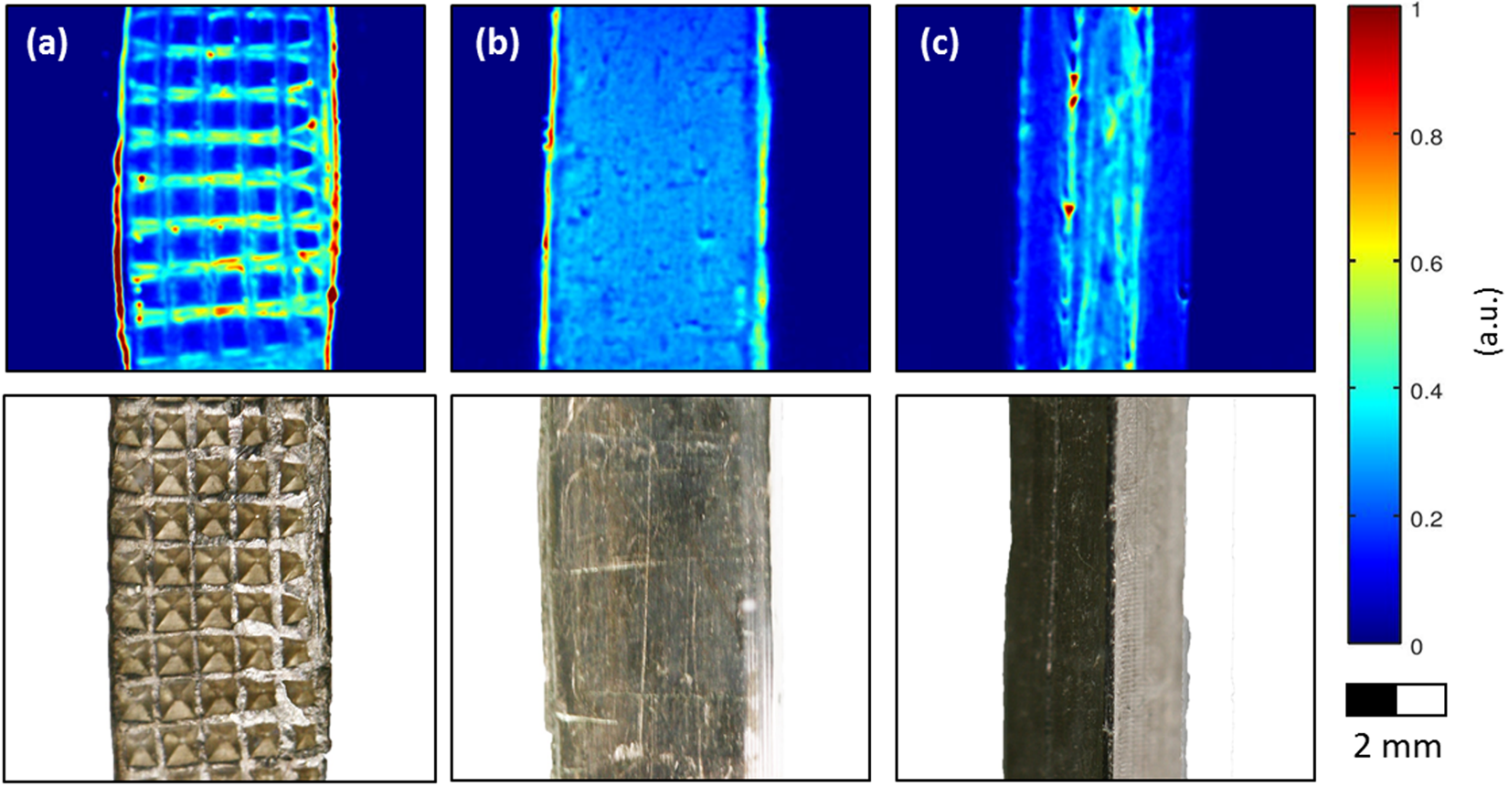
Thick metallic lithium pieces perforated mechanically with different stamps. Top row: EPR images. Bottom row: photographs of the samples. (**a**) Sample with single-sided inward facing pyramidal pattern and a flat surface on the other side. (**b**) Two flat surfaces. (**c**) Lithium piece with a square cross section and a slightly inhomogeneous surface.

To demonstrate CEPRI of metallic lithium with $d\gg \delta$, two different 380 µm thick, flat lithium pieces were investigated. One sample was perforated on one side with a pyramidal structure (Fig. 2a) and compared with an unperforated sample (Fig. 2b). Furthermore, a sample with a quadratic cross section, with the imaging plane parallel to one of the diagonal planes connecting two edges, was analysed (Fig. 2c).

Reference deconvolution did not show any artefacts and the Dysonian phase shift was completely eliminated in the deconvolved projections, which indicates that the CEPR spectrum was invariant at the lithium surface for all three samples. In the case of a multi-component sample with spatially dependent microwave phases or lineshapes, deconvolution using as reference an experimental spectrum with field gradients turned off would not work. Instead, additional projections recorded with varying field gradient strengths would be required^22,23^.

The image of the inward facing pyramids in the sample with one-sided perforation is shown in Fig. 2a. The quadratic valleys are clearly visible and the bars in between, being more exposed to the mw field, show a higher intensity. This suggests that the bars cause shielding and, therefore, lead to the weaker EPR signal of the valleys. A different effect is apparent at the edges. Since images were resolved in two dimensions, the third dimension is represented by a projection of the spin density seen by the microwave onto the image plane. At the edges, the surface exposed to the microwave field is considerably enhanced since also the edge surface perpendicular to the imaging plane contributes to the signal.

The flat sample in Fig. 2b, which was identically prepared yet without surface patterning, shows the same signal enhancement at the edges. Furthermore, signal variations are observed on the flat surface between the edges. While clearly visible in the CEPR image, on the optical image the surface appears to be homogenous. The origin of this contrast is not entirely clear. Local variations of the skin or the spin depth, caused for example by a not perfectly uniform pressure applied during sample preparation or by spatially dependent levels of material impurities, could be a reason.

Considering the image of the square sample in Fig. 2c, the edge shows merely minor, non-uniform enhancement. This is expected, since the same surface area is exposed to the microwave field everywhere, and it confirms the assignment of the enhanced edge signals in Fig. 2a,b.

Since all three samples are much thicker than the skin depth of metallic Li at the microwave frequency used for the EPR experiments, a constant image intensity may be expected for a uniform surface, which is not observed. Instead, it appears that different factors would cause a variation of the Li signal intensity in CEPR images of Fig. 2. On the one hand, local variations of the magnetic microwave field, caused by shielding or eddy current effects, induce a spatially dependent sensitivity of the method. On the other hand, local material variations, which may affect the conductivity, could also cause a contrast. Eventually, in a two-dimensional image, also a variation of the surface area exposed to the microwave field, projected onto the imaging plane, causes a signal variation. Therefore, this analysis is not quantitative regarding the number of electron spins in the sample or on the sample surface^22,23^.

To summarise, the analysis of thick samples allows resolving macroscopic structures, here in the sub-millimetre range, including fine details on their surfaces. However, image contrast is not provided by the local density of Li metal, but by variations of the microwave field caused by the conducting surface features, or by material variations affecting skin or spin depth. Resolution of images with a zero-gradient linewidth of 0.15 mT and an applied gradient of 5 mT cm^−1^ is approximately 50 µm with the use of reference deconvolution. This is consistent with the resolution expected due to self-diffusion of electrons in the material and cannot be improved significantly by optimizing data post-processing despite a high signal-to-noise ratio of the experimental data.

### CEPRI of Thin Sample

**Figure 3:**
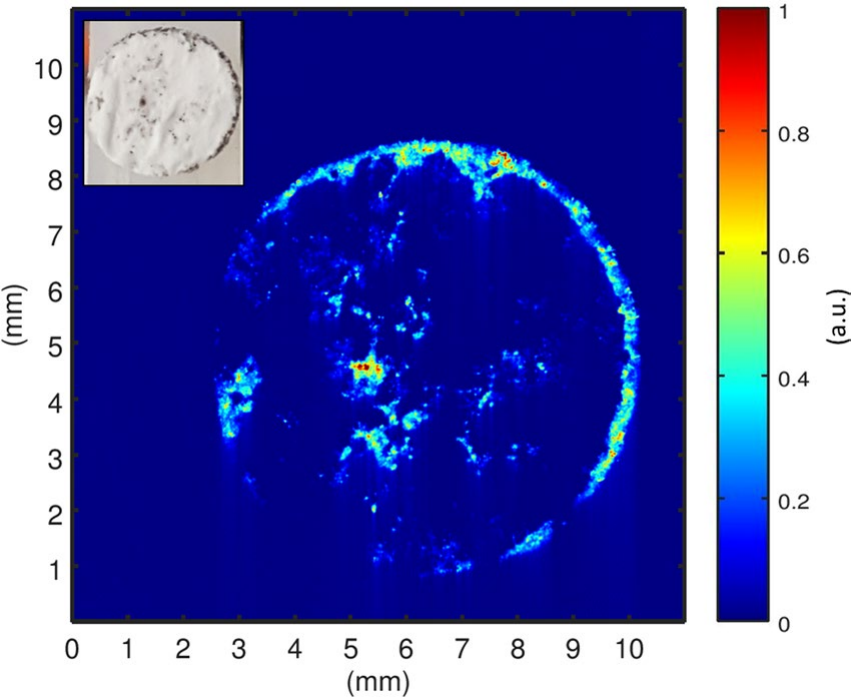
EPR image of lithium dendrites grown in a glass fibre separator with a diameter of 8 mm. The appearance of dendrites is preferably found at the edge of the separator. In addition, an intense dendrite signal is found in its centre. The inset shows a photograph of the separator. The same main features are visible, yet low intensity contributions are more apparent in the EPR image due to the selectivity of the method to lithium metal only.

A battery separator, harvested after cycling a cell with two metallic lithium electrodes, was positioned parallel to the imaging plane inside the EPR resonator. The reconstructed image is shown in Fig. 3. The obtained resolution is variable. While wide features show a resolution of approximately 100 µm, weak features could be resolved with a resolution down to about 8 µm. This behaviour is typical for a diffusion limited resolution with disconnected features of different lengthscale. Since the image was recorded in two dimensions and the separator had an original thickness of 150 µm, the dendrite signal is an accumulation through the height of the separator for each spot in the imaging plane. As a result, a high intensity suggests high concentrations of dendritic lithium.

When comparing the EPR image with a photograph of the separator (Fig. 3, inset), the same main features can be distinguished. Nonetheless, the complementarity of the two techniques is apparent as well. CEPRI provides signal from metallic Li only, hence also weak features can be unambiguously assigned to be originating from dendrites. On the other hand, the origin of weak dark features on the optical image cannot be positively identified, as other types of impurities cannot be excluded. Furthermore, while the optical image is surface sensitive, the EPR image provides the amount of Li metal across the thickness of the separator.

Figure 3 exhibits that dendrite growth is not homogenously distributed across the separator surface, but takes place at preferred sites^39^. Certain spots are more intense, indicating a higher concentration or length of dendritic lithium species. The dendrite distribution provides a hint regarding the current distribution inside the battery, since dendrites are preferably formed at sites with a current density maximum^40^.

Consistent with literature, the electric field in such an electrode arrangement tends to be higher at the edges, which is supported by the observed lithium dendrite growth^41^. In addition, the most intense dendrite spot is found in the centre of the separator. Judging from its amplitude in the CEPR image, this dendrite structure most probably caused the observed short circuit after 200 cell cycles.

Further investigations of cycle dependent dendrite distributions will help to understand the patterns of dendrite growth. In addition, its visualization may aid improving the battery design, including battery-pack shape, cell geometry and active material arrangement^42,43^.

## Conclusion

The capability of CEPRI to investigate metallic lithium samples was demonstrated. Thick lithium samples with textured surface and a battery separator containing dendritic lithium generated by electrochemical cycling were analysed. From these images, structural surface analyses and the determination of conductive lithium distributions are possible, offering new insights for, *e.g*., battery application.

This imaging method shows great potential for imaging of lithium dendrite growth within solid electrolytes, where purely surface sensitive imaging modalities are not sufficient. A quantitative *in situ* application of CEPRI would also be desirable, yet the presence of multiple different Li morphologies and other paramagnetic species greatly complicates this task.

## Methods

### EPR Spectroscopy

CEPR and CEPRI experiments were performed on a Bruker E540 Elexsys X-band spectrometer equipped with a 4108 TMHS resonator. The EPR magnet was equipped with a *y* and a *z* gradient coil, where the *z*-gradient is parallel to the static magnetic field and the *y*-gradient defines a top-bottom view along the resonator. The samples were positioned parallel to the *y*–*z* plane, hence a projection of the signal in *x* direction was recorded.

Imaging of thick lithium samples was done with a gradient *G* = 5 mT cm^−1^. The modulation amplitude was set to 0.03 mT at a frequency of 100 kHz with a microwave power of 0.63 mW. The sweep width was SW = 8 mT with 20 s sweep time, recording *N* = 1024 points. This corresponds to a pixel length of ${\rm{\Delta }}l={\rm{SW}}/((N-1)G)\approx 15.6\,{\rm{\mu }}{\rm{m}}$.

Dendrite imaging was done with a gradient *G *= 2.5 mT cm^−1^, a modulation frequency of 10 kHz at 0.001 mT modulation amplitude and the microwave power set to 1 µW. A single projection was obtained in 240 s with a sweep width of 4 mT, recording 4096 points, which corresponds to ${\rm{\Delta }}l\approx 3.9$ µm. In both experiments 402 projections were recorded.

### Image reconstruction

Image reconstruction was done by first performing a reference deconvolution of the recorded projections using a spectrum that was recorded with field gradients turned off.

Due to the very high signal-to-noise ratio, reference deconvolution was robust using a Gaussian filter with a width of a tenth of a pixel. Therefore, filtering could be excluded as a resolution-limiting factor. Subsequently, a filtered back projection, *i.e*. an inverse Radon transform, with a cosine filter was used to obtain the two-dimensional spatial–spatial image^8,44^.

Processing was performed using GNU Octave (version 4.2.1).

### Sample preparation

Structured lithium metallic samples were prepared with stamps manufactured in-house. Lithium metal was purchased from Sigma–Aldrich with an assigned purity of 99.9%.

A separator containing dendritic lithium was formed in a Swagelok battery cell housing. An axial battery electrode arrangement of 8 mm diameter was used, with two metallic lithium electrodes isolated by a glass fibre separator from VWR with a thickness of 150 µm and electrolyte from Sigma–Aldrich (LP30: 1 M LiPF_6_ in ethylene carbonate and dimethyl carbonate in a 1:1 ratio). The electrodes were fixed between two circular stamps as current collectors. Thereby, metallic lithium was cycled against metallic lithium. Currents of 10 mA were applied for 10 min in each direction for 200 cycles until a short circuit was detected. Afterwards, the separator was extracted without any further cleaning procedure and fixed in a sample holder described elsewhere^45^ under argon atmosphere for EPR imaging.

All lithium samples were handled under argon atmosphere in a glove box and a gas tight container was utilized for EPR measurements.
